# The experience of children with a parent suffering from Amyotrophic Lateral Sclerosis during the COVID-19 pandemic

**DOI:** 10.1038/s41598-021-95338-3

**Published:** 2021-08-06

**Authors:** Ines Testoni, Lorenza Palazzo, Lucia Ronconi, Gabriella Rossi, Jenny Ferizoviku, Jose Ramon Pernia Morales

**Affiliations:** 1 Department of Philosophy, Sociology, Pedagogy and Applied Psychology (FISPPA), University of Padova, 35139 Padova, Italy; 2 Emili Sagol Creative Arts Therapies Research Center, University of Haifa, 3498838 Haifa, Israel; 3 A.I.S.L.A., Italian Association Amyotrophic Lateral Sclerosis, BAOBAB Project Coordinator, 20124 Milano, Italy; 4 Fondazione Mediolanum, 20080 Basiglio (MI), Italy

**Keywords:** Psychology, Health care, Health services, Public health, Quality of life

## Abstract

Children that have a parent with Amyotrophic Lateral Sclerosis (ALS) suffer from the progressive loss of their beloved ones. During the COVID-19 pandemic, the difficulties faced by these children have increased. The study aimed to detect whether there were differences between the minors experiencing a relative’s ALS and the minors with no experience of ALS and it aimed also to detect the impact of COVID-19 pandemic on these minors. The study involved Italian participants, in particular: the target group consisted of 38 children (7–18 years) (T0/T1); the control group consisted of 38 children (9–14 years) (T0 only). The following variables were measured: attachment with the Security Scale (SS), affects with the Positive and Negative Affect Schedule for Children (PANAS-C), behavioural problems with Strengths and Difficulties Questionnaire (SDQ), death representation with Testoni Death Representation Scale for Children (TDRS-C), self-concept with the Multidimensional Self Concept Scale (MSCS), resilience and socio-emotional skills with the Devereux Student Strengths Assessment (DESSA). The results showed higher negative affectivity (*p* < .001), externalising behaviours (*p* < .05), uncertainty in reflective function (*p* < .05) in the target group compared to the control one; after the COVID-19 pandemic minors in the target group showed reduced certainty of mental states (*p* < .05) and interpersonal and scholastic self-esteem (*p* < .05). The impact of ALS on these minors is significant and produces negative affect, externalizing behaviours and uncertainty of mental states. The lockdown situation due to the COVID-19 pandemic has further aggravated minors in their school and interpersonal self-esteem.

## Introduction

Reflective functioning (RF) is the capability of the self to recognise and reflect on one’s mental experience, feelings, thoughts, desires and beliefs, and to construct a representation of one’s psychic life by being aware of the interpersonal implications of one’s mental states^1^. RF is crucial for the ability to regulate affects, the development of self-esteem and beliefs of self-efficacy, for impulse control and the ability to tolerate negative emotions and promote resilience, an essential factor in conditions of prolonged stress, such as a parent’s experiences of illness. In turn, RF significantly depends on the child’s relationship with his/her caregivers^2–4^. RF is related to the neuronal mirroring activated by the attachment experience through parental empathic ability^5,6^; thus, children are involved in the recognition of the responsiveness, which is conveyed primarily by affective facial expressions^7^. The parental reflective functioning (PRF) is the parents’ ability to reflect on their own and their child’s mental states, to understand how these mental states impact behaviour and to guess the child’s intentions^8–10^. The child’s RF develops through his/her interactions with people that are more mature who, in turn, possess appropriate reflexive skills^2,4^. RF works best when the attachment style is more secure. A secure attachment is fostered by the caregivers’ ability to teach the child, through their example, how to manage his/her emotions, especially negative ones, and how to interact with the environment^3,11^. RF permits the development of the self-reflexive ability related to thinking of others in terms of mental states^1,4^. In contrast, insecure attachment is related to the child’s inability to comprehend the world in terms of psychic rather than physical reality^2^. Indeed, when children do not acquire this ability, they are at the mercy of their own and others’ negative emotions; this can result in behavioural and emotional problems because of their vulnerability in facing difficulties^3,4,12^.

Research on PRF has primarily focused on the relationship between mothers and infants. However, PRF may also be important during adolescence, because it helps teenagers cope with their developmental changes and with new social competence, facilitating dialog and coping with the conflict and negative emotions that are characteristic of that age. Although the literature in this field is still scarce, some studies have argued that PRF could be associated with open communication, satisfaction with the parent–child relationship and more positive outcomes to conflicts^13^; other studies have described how adolescents with psychological problems have lower levels of RF^14,15^.

However, no studies in the literature have considered the development of RF in adolescence when PRF is challenged by amyotrophic lateral sclerosis (ALS). ALS is the most common of the motor neuron diseases; it is a progressive neurodegenerative disease that affects both motor neurons and the nerve and spinal cord cells responsible for voluntary muscle movement^16^. Symptoms are characterised by progressive weakness and spasticity in the limbs, difficulty in performing precision tasks, dysphagia, breathing difficulties and speech disorders; when the disease has a bulbar onset, it is often associated with cognitive decline and behavioural problems^17^. The course of ALS is very rapid, and it quickly leads to progressive paralysis. Although the internal organs, the sensory system, eye movement, sphincter control, genital functions and cognitive activities are not affected, especially in the case of bulbar onset, the brain areas for the cognitive and emotional components of empathy are impaired^18–22^. These possible alterations impact facial expressiveness, which is critical to the mirroring process^23–26^ and for RF development^27,28^. Because ALS also immobilises facial motor skills, it is possible to hypothesise that it is difficult for family members, and particularly the youngest among them, to infer the mental condition of the person suffering from this disease.

### Background: anticipatory mourning and ambiguous loss in families with ALS and the COVID-19 pandemic

Daily life is completely disrupted in families with an ALS patient, and this can have negative effects on children^29^. Indeed, the clinical management of ALS is very complex, and it requires both pharmacological and technological devices to improve survival and the possibilities of communicating with others^30^. These aspects imply that every family member of the patient is involved and significant lifestyle changes are required, impacting social relationships and reducing the quality of life^31^. Generally, the primary caregiver is the patient’s partner; however, the patient’s children, who lack sufficient emotional and experiential competences, are involved in dealing with providing assistance (i.e. medications, emotional support, household chores, etc.)^32^. This may result in very stressful reiterate experiences for minors. On the one hand, there is a risk that the development of their identity will be impacted by an excessive sense of responsibility for assisting other people^33^. On the other hand, they have to face emotional experiences^34^, such as ambiguous loss and anticipatory grief^35,36^. Probably one of the most difficult aspects of ambiguous loss is the knotty recognition of the sick parent and of his/her feelings because of the communication difficulties and the paralysis of facial expressions^37–44^. This form of loss can lead to significant emotional distress for minors, who often suffer from anxiety, emotional distress, depression, social isolation, avoidance, somatic disorders, excessive worry about getting sick, sense of guilt, issues related to self-esteem and externalising reactions, such as aggressive behaviour, the propensity to violate the rules and hyperactivity^29,45^.

This already difficult situation was worsened in 2020 by the COVID-19 pandemic, the psychological impact of which has recently been much discussed by emphasising concerns about its potential debilitating consequences on the mental health of youth due to the limitations imposed on their social relationships^46,47^. Indeed, several important theories emphasise the importance of social relatedness as key drivers of human behaviour and optimal psychological functioning for subjective well-being^47,48^. Scholars have focused on the negative effects of the social contact limitations on minors caused by school closures, social distancing and home quarantine^49^. Psychological problems, such as anxiety, depression, irritability, mood swings, inattention and sleep disturbance, have been described as being fairly common among minors^46,50^, emphasising that those who were already disadvantaged before the pandemic were at highest risk of experiencing the worst effects^48,49^. Various types of family difficulties have been considered in the literature, in particular families in quarantine due to the infection or those with economic difficulties or those with family members with psychiatric disorders^47,48^. Unfortunately, there is no literature with respect to the conditions of families with minors in which a parent is already seriously ill, and there is a complete lack of literature regarding minors in families with a parent suffering from ALS. Moreover, in this complex situation in which parent is chronically ill, the attachment relationship between the child and the parent with ALS may also be negatively affected; however, there are no studies that can confirm this hypothesis.

## Method

### Purpose of the present study

The first aim of this study was to detect whether there were differences between the minors experiencing a relative’s ALS (target group) and the minors with no experience of ALS (control group) with respect to the following variables: RF, representations of death, behavioural problems, affect, resilience and socio-emotional skills. The second aim was to evaluate if and how a specific psychological support intervention helped the minors during the first worst months of the COVID-19 pandemic. It was supposed that an intervention focused on the emotional dimension could sufficiently provide support for the minors.

### Psychological intervention with caregivers and minors during the COVID-19 pandemic

This study was begun two months before the COVID-19 outbreak; it aimed to observe the psychological development of RF and evaluate additional variables connected to it among minors who live in a family with a parent suffering from ALS. It was assumed that the development of reflective abilities could help minors cope with ambiguous loss and anticipatory mourning. The study named “Baobab project” was planned with A.I.S.L.A. (Italian Association of Amyotrophic Lateral Sclerosis), a non-profit organisation that offers various forms of support to families with an ALS patient on a national level. In particular, psychological support is guaranteed to all members of the family both via in-person individual and group sessions and through telephone or online sessions. Starting from the results of research already conducted with children of a parent with ALS^29,51^, which highlighted the distress experienced by these minors, and from the literature showing that minors need to process loss and grief with their peers^52^, it has been hypothesised that groups can help the participants process the experience of ambiguous loss and the representation of the ill parent’s mental condition through psychodrama and arts therapy activities.

Unfortunately, immediately after the administration of the T0 assessment, the COVID-19 outbreak occurred, which affected the Italian territory in a very tragic way, especially during the first wave of the pandemic. The initial project was then immediately adapted to the new support needs arising from the disruptions caused by the pandemic. In line with the international indications to support families in difficulty with minors^48,53,54^, the psychological network provided clear information to caregivers with respect how to manage the risks of the pandemic. It was assumed that a good understanding of COVID-19 was important to prevent increasing anxiety and to comply with the measures for containment. Specific information for the caregiver parent was offered with respect to how to explain COVID-19 to minors and how to manage daily life at home during the quarantine and in relation to social distancing and home schooling. The intervention to support minors during the pandemic involved all the family members with a variable number of meetings, differentiated on the basis of specific needs. Each meeting aimed to offer a space for emotional expression in order to improve psychological well-being, contain suffering, foster resilience and improve the quality of life of families, including through the improvement of relationships and communication. The caregivers proved to be extremely cooperative, but they also appeared to be very tired and fatigued due to the compounded difficulty they had to face: assisting their sick relative, working at their job and managing their children during the lockdown. Psychologists had to deal with many difficulties in order to work with the minors, because during the first phases of the pandemic only distance meetings could be offered. This limitation was hardly tolerated by the minors because their school activities were also impacted by distance learning. The main problems that emerged were: suffering for the illness of the relative, the lack of motivation to study, difficulty in managing the gaps in free time, anxiety due to the uncertainty linked to the pandemic and difficulty in bearing the confinement at home. Almost all the minors told the psychologists about the discomfort they felt because their entire constellation of real relationships was converted into virtual ones. However, all the meetings took place via physical distancing (video calls, phone calls, phone messaging).

### Participants

Seventy-six (76) minors were involved in the study. The target group consisted of 38 minors experiencing a relative’s ALS (16 females and 22 males) 7–18 years old [*M* = 11.61, *SD* = 2.97]). 16 children (42.11%) attended primary school, 9 (23.7%) middle school, and 13 (34.2%) high school. All the participants were Italian; 36 were Catholic and 2 were Muslim. 15 participants (39.5%) came from a family with medium/low socio-economic status, 19 (50%) medium and 4 (10.53%) medium/high.

The control group consisted of 38 minors with no experience of ALS (16 females and 22 males) 9–14 years old [*M* = 11.39, *SD* = 1.82]) selected from 210 students (120 females, 90 males) 9–14 years old [*M* = 11.17, *SD* = 1.33] from two schools in Northern Italy, in order to obtain a control group with the same number of participants as the target group and without differences in gender and age.

The target group of minors was collected with a purposive sampling, in particular an homogeneous sampling: the psychologists of different offices of the non-profit association involved in the project proposed participation to families who respected the criterion of having a parent with ALS who had one or more children aged 7 to 18. The families were explained that the aim was to understand the needs of the minors and how to help them and that participation was independent of the services received by the association.

For the control group, the search of the participants started from some schools known by the researchers and then proceeded with snowball sampling based on the willingness of teachers and parents to participate in the research.

The participants and their parents were informed of the objectives and procedures of the research, and the confidentiality of their answers was guaranteed. We obtained informed consent from all the participants and their parents before administering the questionnaires. The study followed the American Psychological Association Ethical Principles of Psychologists and Code of Conduct and the principles of the Declaration of Helsinki. It received research ethics approval from the Health Sciences and Science Research Ethics Committee of the University of Padua (reference: C3DD8C5FCE1C26C7E80954B4EC34DC16).

### Procedures

**Figure 1:**
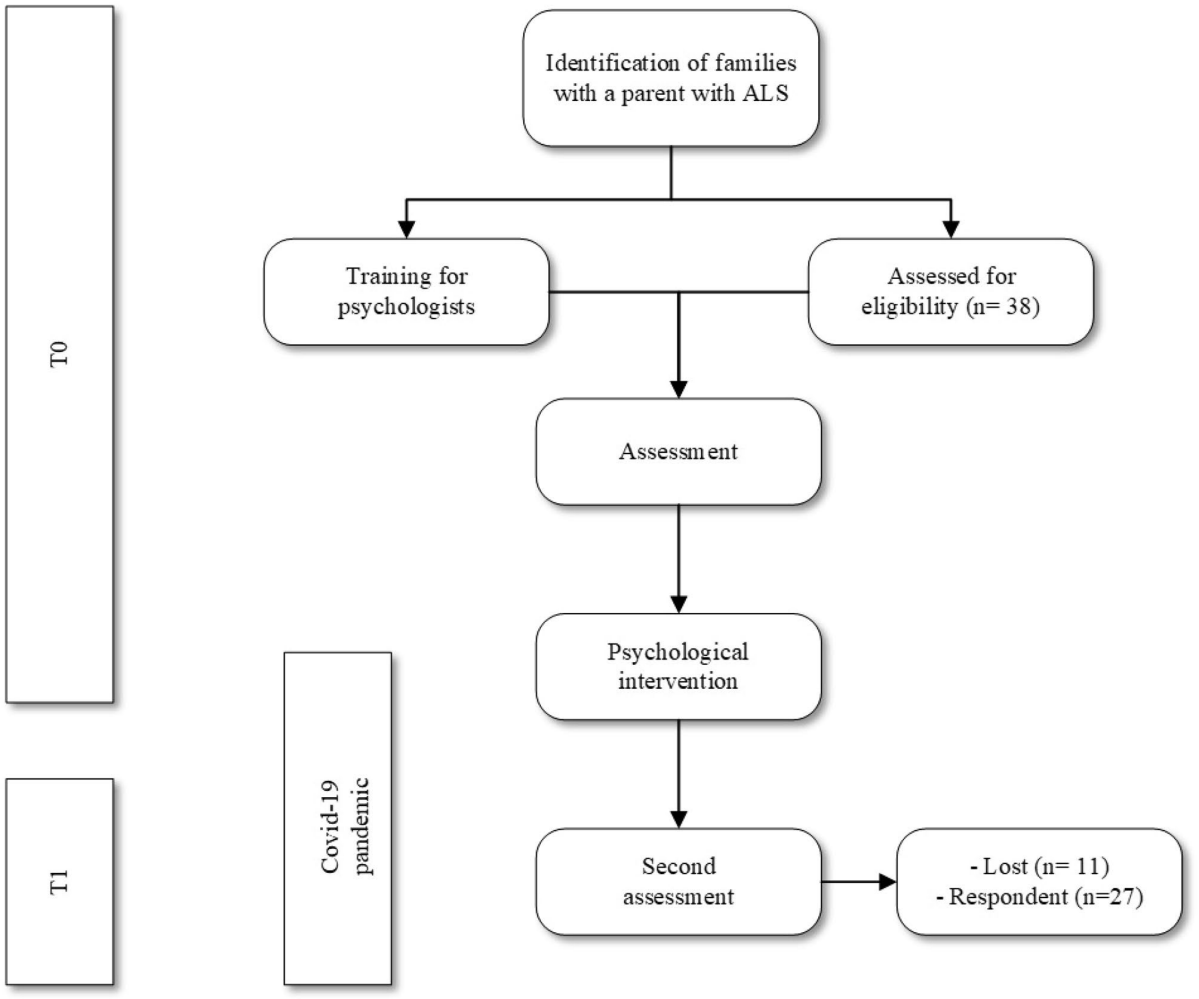
Flow chart of the study protocol.

The families that participated in this study were helped by a non-profit organisation supported by 11 psychologists belonging to different offices of the association throughout Italy (specifically, five from the northern part of Italy, two from the central part and four from the southern part). The families were proposed to participate in the longitudinal surveys in order to understand the needs of the minors and how to help them. Before the project, the psychologists took part in specific training held at the University of Padua on the RF, the management of experiences related to anticipatory grief and ambiguous loss and transformative learning. The latter is a construct linked to the RF—as an indication of the extent of the maturation of the reflective self that was analysed in this study—and it comes from the studies conducted by Mezirow^55^. According to the concept of transformative learning, people learn and grow when their perspectives of meaning for interpreting their experiences change following a critical and stressful event. This results in a critical reflection of the assumptions taken for granted, and it leads to new ways of thinking, influencing personal changes in daily life. Transformative learning provides a high level of reflectivity that can be developed and strengthened with specific support strategies relying on the dimensions that support it^56^. The psychologists administered the questionnaires to each of the children and adolescents in the target group before the support interviews and 7 months later at the end of the psychological support intervention. The administration lasted about 1 h. The intervention began in person in January 2020 in the offices of the association, and it was interrupted due to the national lockdown that took place in Italy during the spring of 2020 due to the COVID-19 pandemic. According to the families, some of the children and adolescents have resumed the intervention with the psychologists electronically on Zoom, while others restarted it as soon as it was possible to reopen the association’s offices beginning in May 2020. The control group was involved following an explicit request from researchers in two schools in Northern Italy that were willing to participate after presenting the project to the students’ families. A researcher administered these protocols with the control group within the students’ classes and he remained available in the classroom during the administration. It took about 40 min, on average, for the participants to fill in the questionnaires. The flowchart below (Fig. 1) summarizes the phases of the research project protocol and the procedures of the study.

### Protocol for target group

Participants of target group completed, before and after the intervention, all the following instruments: the Security Scale (SS), the Positive and Negative Affect Schedule for Children (PANAS-C), the Strengths and Difficulties Questionnaire (SDQ), the Reflective Functioning Questionnaire (RFQ), the Testoni Death Representation Scale (TDRS-C), the Multidimensional Self Concept Scale (MSCS) and the Devereux Student Strengths Assessment (DESSA).

### Protocol for control group

Participants of control group completed only the following instruments: the PANAS-C, the SDQ, the RFQ and the TDRS-C. This protocol variation was adopted to make the administration shorter and suitable for the time allowed by the participating schools, and the DESSA presupposed an additional step of completion by the parents, which was not possible to carry out.

### Measures

The questionnaires for the target group included the following instruments: the Security Scale (SS), the Positive and Negative Affect Schedule for Children (PANAS-C), the Strengths and Difficulties Questionnaire (SDQ), the Reflective Functioning Questionnaire (RFQ), the Testoni Death Representation Scale (TDRS-C), the Multidimensional Self Concept Scale (MSCS) and the Devereux Student Strengths Assessment (DESSA).

The SS^57,58^ is a self-report questionnaire that is used to assessed perceived security and attachment. It is divided into two parts, one referring to the mother and one to the father, each consisting of 15 questions. The child/pre-adolescent is asked to indicate which of the two situations that are described feels most similar to him/her by choosing between two options: “True enough for me” or “Very true for me”. Cronbach’s alpha for perceived security is 0.70 before intervention and 0.71 after intervention for mother and 0.85 and 0.91 for father.

The PANAS-C^58,59^ is a self-report questionnaire that consists of 30 adjectives divided into 15 positive terms (positive affect [PA] scale) and 15 negative terms (negative affect [NA] scale) in which the respondent must indicate the intensity with which he/she feels these emotions by choosing from five options where 1 corresponds to “very little” and 5 corresponds to “a lot”. The children’s version has been adapted from the adult version, and it has good internal consistency and convergent and discriminating validity in comparison to other self-report measures of positive affect and negative affect for children^60^. Cronbach’s alpha for positive affect is 0.92, before and after intervention, and 0.91 for target and control group together; Cronbach’s alpha for negative affect is.91, before and after intervention, and 0.90 for target and control group together.

The SDQ^61^ is a self-report multiple choice questionnaire in which people are asked to choose their response based on a Likert scale ranging from 1 to 3 (“Not true”, “Partially true” and “Absolutely true”), from a list of 25 statements. The SDQ investigates behavioural problems by considering five areas: emotional symptoms (EMO), behavioural problems (COND), inattention/hyperactivity (HYPER), peer problems (PEER) and prosocial behaviours (PROS). HYPER and COND represent the subscales of externalising problems, while EMO, PEER and PROS represent the subscales of internalising problems. All the scales are added together to generate a Total Difficulty score for each minor. Regarding the percentiles that represent the cut-off scores to consider the difficulties of children and young people as problems and to divide them into the categories, we used “normal”, “sub-clinical” and “clinical”, from the Italian validation, as the reference^62^. The SDQ was used in the Italian version freely available on the official website of the questionnaire and the scoring was done manually. Cronbach’s alpha values for EMO are 0.79 before intervention, 0.77 after intervention and 0.75 for target and control group together; for COND are 0.49 before intervention, 0.67 after intervention and 0.60 for target and control group together; for HYPER are 0.77 before intervention, 0.63 after intervention and 0.70 for target and control group together; for PEER are 0.76 before intervention, 0.52 after intervention and 0.70 for target and control group together; for PROS are 0.70 before intervention, 0.70 after intervention and 0.65 for target and control group together; for Externalising Problems are 0.81 before intervention, 0.77 after intervention and 0.80 for target and control group together; for Internalising Problems are 0.88 before intervention, 0.77 after intervention and 0.81 for target and control group together and for total score are 0.91 before intervention, 0.81 after intervention and 0.88 for target and control group together.

The RFQ^63^ is a self-report questionnaire used to evaluate of the psychological processes underlying the ability to mentalise. The scale was created for adults, but it is also used with teenagers^64^. The short version was chosen for this project; it consists of eight items divided into two subscales: Certainty (RFQ_C) and Uncertainty (RFQ_U) with respect to one’s own and others’ mental state. The RFQ requires the respondent to choose a number between 1 and 7 to express how much he/she agrees or disagrees with each of the statements, where 1 corresponds to “Strongly disagree” and 7 corresponds to “Strongly agree”. The average scores on both scales indicate a good mentalisation ability; certainty scores that are too high indicate a lack of awareness in believing that the mental processes of others are not completely knowable and remain partially incomprehensible (hyper-mentalisation); uncertainty scores that are too low indicate poor mentalisation skills, which is known as hypo-mentalisation^65^. Cronbach’s alpha for RFQ_C is 0.75 before intervention, 0.87 after intervention and 0.71 for target and control group together; Cronbach’s alpha for RFQ_U is 0.47 before intervention, 0.57 after intervention and 0.47 for target and control group together.

The TDRS-C^66^ is a self-report questionnaire consisting of six items ranked using a Likert scale ranging from 1 to 5, where 1 indicates total disagreement with the statement and 5 indicates total agreement. In the version for children and pre-adolescents used in this study, four items are used. The questionnaire allows for detecting whether a person represents death as a passage (items 1, 5 and 6) or as annihilation (items 2, 3 and 4). A higher score refers to a representation of death as annihilation, while a lower score refers to a representation of death as a passage. Cronbach’s alpha is 0.66 before intervention, 0.50 after intervention and 0.75 for target and control group together.

The MSCS^67,68^ is a questionnaire that allows for the precise measurement of self-esteem based on developmental age, in its many dimensions. It evaluates six areas of general self-esteem: interpersonal area, school area, family area, body area, area of emotional competence and mastery of the environment. For the 150 statements presented, the respondent can choose an answer ranging between “Absolutely true”, “True”, “Not true” and “Not absolutely true”. From these six areas, an overall self-esteem score is obtained. Cronbach’s alpha values for interpersonal area are 0.92 before intervention and 0.88 after intervention; for school area are 0.95 before intervention and 0.92 after intervention; for family area are 0.96 before intervention and 0.95 after intervention; for body area are 0.91 before intervention and 0.90 after intervention; for emotional competence area are 0.94 before intervention and 0.92 after intervention; for mastery of the environment area are 0.88 before intervention and 0.90 after intervention and for total self-esteem are 0.98 before intervention and 0.97 after intervention.

The DESSA^69^ consists of 72 items that are used to assess the resilience and socio-emotional skills of children from the age of kindergarten on. In particular, it analyses self-awareness, self-regulation, social awareness, interpersonal skills, a sense of responsibility, decision-making capacity, purpose-oriented behaviour and optimistic thinking. It is built to be filled in by the parent caregiver and/or a teacher who, thinking about their child in the last 4 weeks, has to choose an answer ranging between “Never”, “Rarely”, “Sometimes”, “Often” and “Very often”. Cronbach’s alpha for total socio-emotional competence is 0.97 before intervention and 0.98 after intervention.

### Statistical analysis

Preliminary descriptive analysis of all the variables indicated no relevant departure from normal distribution except for the two RFQ factors. A square-root transformation was applied to normalise the distribution of these variables. Independent samples t-tests were used to analyse the differences between the target group and the control group. Paired samples t-tests were used to analyse the differences between the pre-test and post-test administration for the target group. We used Cohen’s *d*^70^ to estimate the effect sizes of all the mean differences.

## Results

Children experiencing a relative’s ALS exhibited a significantly higher negative affect and a lower positive affect than their peers with no experience of ALS (Table 1). The effect size of these mean differences is large for negative affect (d = 0.87) and medium for positive affect (d =  − 0.52). The children in the target group also exhibited significantly more externalising problems, and in particular problems with hyperactivity and inattention, than the children in the control group. Both these mean differences have a medium effect size (d = 0.46 and d = 0.67, respectively). Moreover, the children in the target group showed significantly less certainty about mental states than the children in the control group with a medium effect size for mean difference (d =  − 0.55). No differences were found for death representation between the two groups.

**Table 1:** Descriptive statistics of study variables for target group and control group with t-test results and Cohen’s *d***.**

Measures	Target group (*N* = 38)		Control group (*N* = 38)		*t* (74)	Cohen's *d*
	*M*	*DS*	*M*	*DS*		
RFQ—Certainty about mental states^1^	0.77	0.47	0.99	0.34	− 2.39*	− 0.55
RFQ—Uncertainty about mental states^1^	0.55	0.39	0.72	0.37	− 1.95	− 0.45
TDRS—Death representation as annihilation	2.60	0.68	2.42	1.07	0.84	0.19
PANAS—Positive Affect	52.87	12.07	58.11	7.38	− 2.28*	− 0.52
PANAS—Negative Affect	34.32	11.52	25.68	7.92	3.81***	0.87
SDQ—Emotional Symptoms	3.84	2.88	2.92	2.43	1.51	0.35
SDQ—Conduct Problems	2.45	1.88	2.16	1.94	0.66	0.15
SDQ—Hyperactivity–Inattention	4.55	2.65	3.08	1.67	2.90**	0.67
SDQ—Peer Problems	1.71	2.08	1.45	1.37	0.65	0.15
SDQ—Prosocial Behaviour	8.00	1.68	7.47	1.66	1.38	0.32
SDQ—Internalizing Problems	5.55	4.60	4.37	3.18	1.30	0.30
SDQ—Externalizing Problems	7.00	4.27	5.24	3.27	2.02*	0.46
SDQ—Total Problems	12.55	8.22	9.61	5.03	1.89	0.43

^1^A square root transformation was applied to normalize the distribution of the two RFQ factors.

*p < .05; **p < .01; ***p < .001.

Only 27 of the 38 children in the target group had no missing values at the post-test administration, and could be considered for the changes over time examination. No significant changes were observed in any of the study variables except for the certainty of mental states and self-esteem related to two areas: interpersonal relationships and scholastic success (Table 2). These mean differences indicate a decrease in the scores from the pre-test to the post-test, with a medium effect size (d = 0.41, d = 0.40 and d = 0.40, respectively).

**Table 2:** Descriptive statistics of study variables for target group at the pre-test and the post-test administration with t-test results and Cohen’s *d***.**

Measures	Pre-test (*N* = 27)		Post-test (*N* = 27)		*t* (26)	Cohen's *d*
	*M*	*DS*	*M*	*DS*		
RFQ—Certainty about mental states^1^	0.65	0.46	0.46	0.50	2.15*	0.41
RFQ—Uncertainty about mental states^1^	0.67	0.34	0.78	0.36	− 1.20	− 0.23
TDRS—Death representation as annihilation	2.70	0.70	2.81	0.47	− 0.88	− 0.17
PANAS—Positive Affect	49.26	11.37	47.70	10.08	0.73	0.14
PANAS—Negative Affect	35.41	11.76	37.56	10.40	− 1.03	− 0.20
SDQ—Total Problems	13.96	8.30	14.37	6.65	− 0.39	− 0.07
SS—Attachment security mother	45.96	5.52	46.07	5.52	− 0.11	− 0.02
SS—Attachment security father	43.70	6.84	43.85	8.70	− 0.16	− 0.03
MSCS—Interpersonal Relationships	77.78	9.40	75.51	9.01	2.10*	0.40
MSCS—Competence Environmental Control	72.11	8.02	72.30	9.06	− 0.14	− 0.03
MSCS—Emotionality	72.48	11.13	68.64	10.90	1.89	0.36
MSCS—Scholastic Success	72.69	13.01	69.10	10.29	2.09*	0.40
MSCS—Family Life	83.66	9.78	82.63	9.89	0.55	0.11
MSCS—Body Identity	74.11	9.82	71.49	10.07	1.32	0.25
MSCS—Total self-esteem	452.78	49.76	439.67	42.12	1.62	0.31
DESSA—Total socio-emotional competence	161.15	40.03	169.89	43.99	− 1.43	− 0.27

^1^A square root transformation was applied to normalize the distribution of the two RFQ factors.

*p < .05; **p < .01; ***p < .001.

## Discussion

Initially, the study had begun to implement intervention strategies to help the children of ALS patients cope with their ambiguous loss by working on the RF with psychodrama and arts therapy to process the representation of the mental condition of their ill parent. The intervention was then modified because, after the T0 detection, the COVID-19 pandemic exploded and everything was diverted to managing its effects during the period of the lockdown when Italy was the country that was most plagued by the pandemic worldwide after China. This was a time of great stress for everyone, and particularly for families with school-aged children. During the lockdown, minors could not attend in-person classes at school and they could not engage in the normal activities of playing and sharing experiences with their peers. In particular, it is important to consider that additional factors weighed on the families of the participants; during the pandemic, the course of ALS still proceeded to worsening the condition of the sick parent and the difficulties of the caregiver because they could not even rely on the social support system to manage their children. The study paid particular attention to RF, the representations of death, behavioural problems, affect, resilience and socio-emotional skills. In T0, as hypothesised, the study’s results indicate that the sense of well-being was lower for children with a relative suffering from ALS than children with no experience of ALS, as already observed in the literature^72,73^. However, among the participants of the target group, despite their more difficult initial state, their psychological condition did not worsen over time. In particular, the children’s perceived security towards their parents did not decrease, and there was no increase in behavioural difficulties as measured with the SDQ, from either an internalising point of view or an externalising point of view. Since the health condition of the ALS parent inevitably worsened, it is possible to infer that the caregiver parents were able to contain the negative effects caused by both ALS and the restrictions imposed by COVID-19, and it did not worsen their relationship with their children. It is also possible to hypothesise that the help offered by the association to caregivers and minors during the pandemic was able to adequately support these families. In particular, it is important to emphasise that, in T0, the children in the target group had significantly higher scores on the HYPER scale than the children in the control group, as already discussed in the literature about minors facing the chronic disease of a parent^29,33,51,72^. Because the lockdown drastically reduced the participants’ opportunities to vent their need to act in the world (closure of gyms, pools, playgrounds, youth meeting places, oratories, etc.), the fact that this aspect did not worsen allows us to assume that the psychological support for the caregivers and children contained the psychosocial distressing effects of the COVID-19 pandemic. However, the scores of two variables decreased: 1) self-esteem related to two interpersonal relationships and scholastic success and 2) certainty of mental states. The worsening of the certainty of mental states is linked to the duration of the lockdown, when the aspects related to interpersonal and school relationships came to a sudden halt and were inevitably negatively influenced by social isolation and distance learning. With respect to the first worsened dimension, the MSCS questionnaire is based on Bracken et al.’s^74^ view of the multidimensional concept of Self, in which the six domains that make up the concept of Self (interpersonal, environmental control competence, emotional, scholastic, familial and bodily) contribute to an individual’s self-esteem. These domains are separate not from each other; rather, they are overlapping and interconnected, constituting a hierarchical model that is dependent on contextual factors. The dimension of academic success is better known in the literature as the Academic Self Concept, and it can be defined as each individual’s perception of his or her overall abilities in the school context^75,76^. The self-image that children and adolescents construct at school affects their entire personality; those who possess good academic self-esteem are more likely to perform well in school, experience good educational and behavioural outcomes, exert more effort in schoolwork, achieve more success and have more motivation and higher academic and career aspirations^77^. Furthermore, the dimension of self-esteem related to social and interpersonal skills provides a measure of how children evaluate their ability to interact with others in different contexts^78,79^. The fact that this area has worsened among our participants highlights how important it is for students to experience in-person schoolwork with their peers. Therefore, the loss of confidence with respect to this area requires targeted interventions and social strategies that are different from those adopted to contain the COVID-19 pandemic. RF was the second area that worsened. Indeed, reflective skills are also linked to the relational and interpersonal aspects that have been affected by the total lockdown and the social isolation. Certainty of mental states is an inherent part of an individual’s genuine capacity for mentalisation, being aware of his/her mental states and those of others and even admitting that there is a margin of inscrutability. The lack of this balance is referred to as hyper-mentalisation when overly complex and unrealistic models are imagined, or hypo-mentalisation when the ability to represent one’s own and others’ mental states is reduced. Hypo-mentalisation is correlated with imaginative poverty and reduced communication and social skills as seen in autism spectrum disorders or borderline personality disorders, and alexithymia, major depression, impulsivity and poor anger control. A high level of uncertainty of one’s own and others’ mental states does not allow individuals to accurately interpret and understand social experiences, thus making them more vulnerable to psychopathological onset^62–64^.

Currently, it is impossible to say whether this worsening is due to the worsening health condition of the parent with ALS, which could have made the representation of his/her mental condition even more difficult, or to the condition of social isolation and detachment from peers brought on by the pandemic. However, this research study highlights that, together, these two factors can cause that effect. Future research could investigate if the same problem occurs in a normal socialisation condition. In this case, it could be considered that the worsening of the ALS, and therefore the inexpressiveness of the parent’s face, has a negative impact on the development of RF in minors. A final point about RF and its relationship to attachment and spirituality must be made. The spiritual dimension allows one to represent the afterlife and thus the continuity of the attachment relationship with the deceased loved one. This implies the possibility of seeing death as a passage rather than viewing it as annihilation. There are not yet studies that relate RF with spirituality and the possibility of maintaining a link with the attachment figure by imagining the possibility of a relationship with the deceased, representing his/her mental state. Certainly, some studies done on continuing bonds have shown how these allow an individual to better overcome the suffering of mourning^80,81^. Originally, the project planned to develop representations of death by expanding the horizon to include reflection on transcendence to allow minors to think that death does not mean the absolute loss of a loved one, as death education experiences have shown^82–84^.

Unfortunately, it was not possible to do this type of elaboration due to the urgency created by the pandemic. In fact, with respect to the representation of death, no significant modifications were observed. The participants always maintained an average score, tending more toward a representation of death as annihilation, but this did not change significantly over time. This result is positive, because the representation of death as annihilation is usually associated with greater anxiety and fear of death, whereas a representation of death as a passage is associated with a more serene attitude towards this topic and less alexithymia, fear of death and generalised anxiety^83,86^. However, it is necessary to emphasise that minors usually represent death as a passage, and they think of an afterlife as representing the continuity that exists beyond bodily death^87–89^. This finding shows that the children in our target group represented death as annihilation to a significantly greater extent than the children in the control group in T0. The fact that this representation was consistent over time suggests that the influence of the relationship with the ASL parent influenced the children’s thoughts about death in a nihilistic way. However, in the literature there is a wide consensus that the loss, grief, bereavement and mourning caused by the severe illness and death of a parent is very stressful for minors and that a good relationship with peers increases their self-esteem and helps them cope with suffering from loss^90,91^. Thus, the pandemic confirmed the central role of socialisation in the lives of minors, especially when they have to manage and compensate for their parents’ difficulties.

## Conclusion

The present study aimed to observe the changes among children of a parent with ALS, evaluating the effectiveness of a specific psychological support intervention with these minors during the first lockdown due to the COVID-19 pandemic. In particular, it was possible to observe the consequences that the reduction of the children’s contact with their peers at school had on their well-being and their reflective and mentalising abilities. It was possible to observe that the impact of ALS on these minors is indeed important, especially with respect to negative affectivity, the presence of externalising behaviours and less certainty about their own mental states. The psychological intervention aimed at supporting the children from an emotional point of view was effective in not increasing negative affectivity, not eliciting behavioural problems and not decreasing their resilience and social-emotional capacities. However, isolation from their friends at school negatively affected their school and interpersonal self-esteem and worsened their certainty regarding their own and others’ mental states. This finding has important clinical implications with respect to the need to prepare and promote future supportive interventions that protect these children from such difficulties.

## Limitations and future developments

This research study has some limitations. First, it was not possible to administer all the questionnaires to the participants in the control group because some of the questionnaires were more complex and required either more time to complete (SS) or the presence of a parent (DESSA), which are requests that the schools could not meet. A further limitation was that it was not possible to carry out the second set of questionnaires with the participants in the control group at T1 because the schools were unwilling to proceed due to the serious difficulties caused by COVID-19. Moreover, the measures carried out did not allow to observe the changes following the psychological intervention, also due to the fact that between the t0 and t1 the Covid-19 pandemic variable intervened and modified further the situations of these minors.

However, this study’s findings highlight the suffering of young people at this time of the pandemic and it represents a significant contribution from which to understand the consequences we will see in the near future and to implement preventive actions to address them. Future research could also investigate the variations of these functions in adolescents with healthy parents in order to understand if the limitation of contact with their peers has repercussions on their RF.

With regard to the study’s target group, future studies could also develop a qualitative survey with in-depth interviews to investigate the anxieties and fears related to the mental state of the sick parent. It would be very important to develop the research by conducting a more in-depth investigation of the RF of the caregiver parent and cross-referencing data between the parents and their children.
